# Optimal extraction conditions and quantification of lignan phytoestrogens in cereal grains using targeted LC-MS/MS

**DOI:** 10.3389/fnut.2024.1409309

**Published:** 2024-06-11

**Authors:** Yoonjeong Kim, Heon-Woong Kim, Jeehye Sung, Younghwa Kim

**Affiliations:** ^1^Department of Food Science and Biotechnology, Kyungsung University, Busan, Republic of Korea; ^2^National Institute of Agricultural Sciences, Rural Development Administration, Wanju, Republic of Korea; ^3^Department of Food Science and Biotechnology, Andong National University, Andong, Republic of Korea; ^4^Food and Life Science Research Institute, Kyungsung University, Busan, Republic of Korea

**Keywords:** lignans, LC-MS/MS, grains, RSM, optimal extraction

## Abstract

Lignans are phytoestrogens found in various forms such as glycosides, ester-linked oligomers, and aglycones in a variety of foods, including soy products, legumes, grains, nuts, vegetables, and fruits. This study aimed to optimize the extraction of lignans from cereal grains using response surface methodology (RSM). Lignans, including secoisolariciresinol (Seco), matairesinol (Mat), pinoresinol (Pin), lariciresinol (Lar), and syringaresinol (Syr), were quantified using high-performance liquid chromatography–tandem mass spectrometry. A Box–Behnken design was employed to determine the optimal values for three extraction parameters: temperature (X1: 20°C–60°C), methanol concentration (X2: 60%–100%), and extraction time (X3: 30–90 min). The highest lignan contents were obtained at X1 = 44.24°C, X2 = 84.64%, and X3 = 53.63 min. To apply these experimental conditions to the actual experiment, the optimal conditions were slightly adjusted to X1 = 40°C, X2 = 80%, and X3 = 60 min. The predicted results closely matched the experimental results obtained using the modified optimal extraction conditions. The highest lignan content found in barley sprouts (85.930 μg/100 g), however, most grains exhibited relatively low concentrations of lignans. These findings provide valuable insights into the lignan content of grains and contribute to the generation of reliable data in this field.

## 1 Introduction

Lignans are phenylpropanoid dimers, where the phenylpropane units are linked by the central carbon of their side chains (1). Lignans, including secoisolariciresinol (Seco), matairesinol (Mat), pinoresinol (Pin), medioresinol (Med), lariciresinol (Lar), syringaresinol (Syr), sesamin (Ses), 7′-hydroxymatairesinol (HMR), and isolariciresinol, are widely distributed in the bark, bulbs, leaves, seeds, and stems of plants (2, 3). The compounds occur mainly in the glycoside, ester-linked oligomer, and aglycone forms (4, 5). Lignans are known as potential antioxidants along with phenolic compounds such as flavonoids and phenolic acids (6, 7). Some of the plant lignans including secoisolariciresinol diglucoside, Mat, Pin, and Lar are deglycosylated, dehydroxylated, demethylated, and converted by intestinal bacteria to the mammalian lignans enterodiol and enterolactone and then absorbed through the colon (8–10). Moreover, lignans, referred to as phytoestrogens, bind to estrogen receptors and may exhibit both estrogenic and anti-estrogenic effects, affecting conditions such as menopause, cardiovascular disease, and cancer (11, 12). Recent studies demonstrated the bioaccessibility and bioavailability of plant lignans during digestion and fermentation in foodstuffs (13, 14). Grain such as wheat, corn, rice, oats, millet, barley, spelt, and rye, are commonly consumed worldwide (15, 16). Various studies have investigated the absorption and role of dietary lignans from grains (17–19). Moreover, Nørskov et al. (20) have investigated the LC–MS/MS method of the free and bound form of plant lignans and enterolignans after consumption of cereal-based diets. Previous studies have shown the lignan content in seeds, vegetables, and fruits (21). Smeds et al. (22) evaluated that lignans, including HMR, Seco, Mat, Lar, Pin, Med, and Syr, were quantified in wheat, oat, and rye. Thompson et al. (23) reported the isoflavone, lignan, and coumestan contents in various foods (e.g., soy products, legumes, nuts, grains, vegetables, and fruits) commonly consumed in Canadian diets. Lignan contents in seeds, such as sesame seeds and flaxseeds have been reported (24–26), but research on lignan contents in cereal grain and their products has been limited.

**Figure 1:**
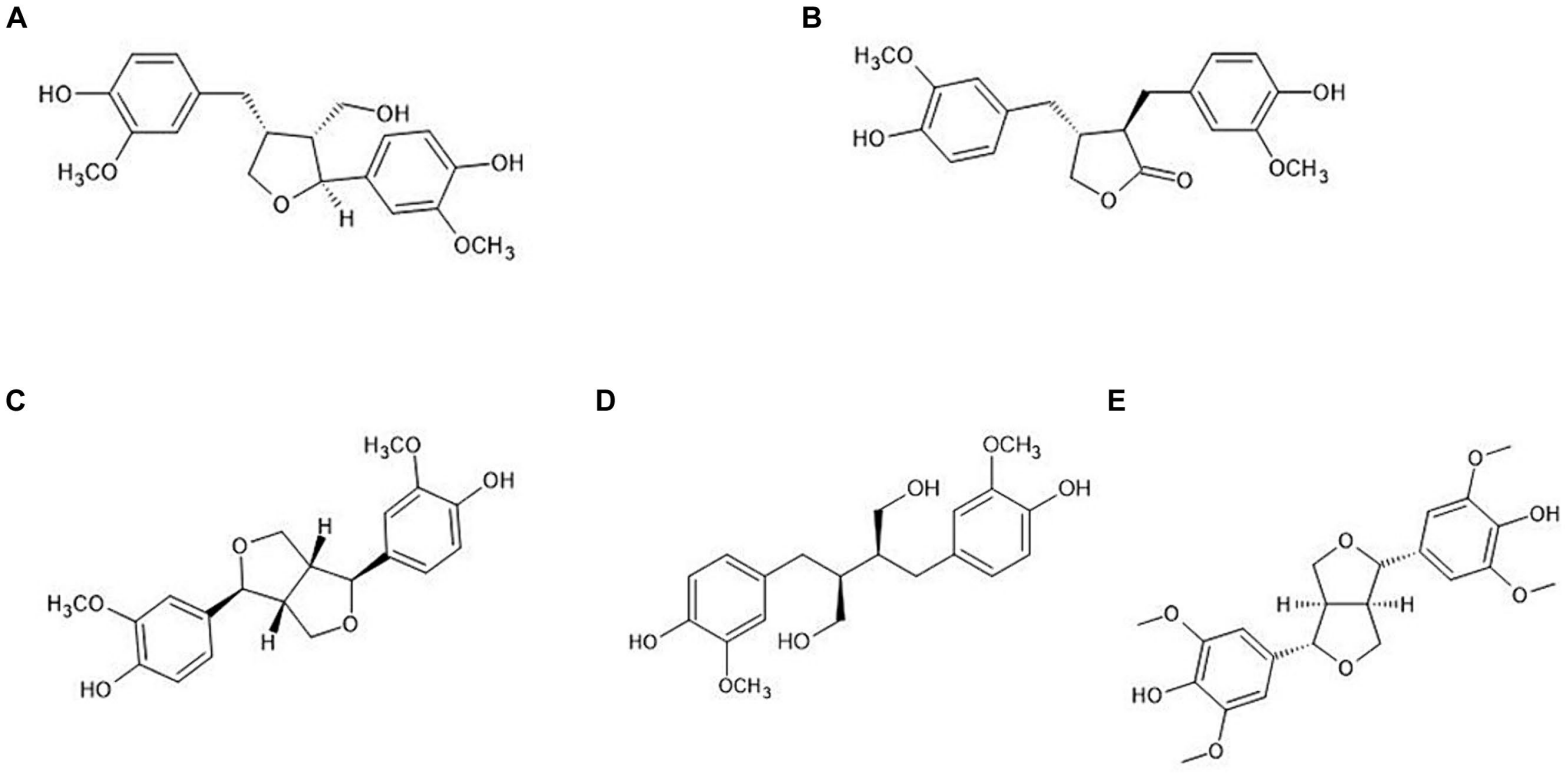
Chemical structure of plant lignans. **(A)** Lariciresinol; **(B)** matairesinol; **(C)** pinoresinol; **(D)** secoisolariciresinol; **(E)** syringaresinol.

Various extraction methods of lignans in plants have been reported, such as ultrasound-assisted extraction, Soxhlet extraction, reflux extraction, and microwave-assisted extraction, using organic solvents including methanol, ethanol, and ethyl acetate (27–29). Additionally, hydrolysis methods have been studied to break the glycoside linkages of lignans, such as enzymatic, acidic, and alkaline hydrolysis (24, 30, 31). Liquid chromatography combined with mass spectrometry is preferred for metabolite identification and quantitative research in foods and drugs due to its sensitivity, fast analysis times, simplicity of sample preparation, and mass accuracy (32–34). López et al. (35) reported that Pin, hydroxy-Pin, and acetoxy-Pin, found in olive oil, were quantitatively analyzed by high-performance liquid chromatography mass spectrometry (HPLC–MS/MS). Moreover, Kuhnle et al. (36) reported that Seco and Mat were simultaneously analyzed in vegetables using HPLC–MS/MS. Response Surface Methodology (RSM) is a mathematical and statistical technique for determining the optimal values to describe the relationship between independent variables and responses (37). Currently, RSM is applied in the food and pharmacological industries to optimize compound extraction from various foods (38, 39). Zhang et al. (40) used RSM to optimize the extraction of lignans from flaxseed, and Zhao et al. (41) used RSM to optimize schizandrin and schisandrol B extraction from *Schisandra chinensis* using accelerated solvent extraction. A previous study evaluated the influence of antioxidants, including polyphenols and vitamin B2, in ultrasound-assisted extraction using the response surface method with Box–Behnken design (42). However, only a limited number of studies have reported on extraction methods and simultaneous analysis of lignans such as Lar, Mat, Pin, Seco, and Syr found in cereal grain. This study aimed to determine the content of five lignans (Figure 1; Lar, Mat, Pin, Seco, and Syr) in cereal grains and their products, and to assess the optimal extraction conditions of lignans using RSM.

## 2 Materials and methods

### 2.1 Standards and reagents

All the reagents were of analytical and HPLC grade. Syr (≥98%) was obtained from ChemFaces (Wuhan, China). Seco (≥95%), Mat (≥85%), Lar (≥95%), and Pin (≥95%) were purchased from Sigma-Aldrich (St. Louis, Mo, United States). Acetonitrile (ACN) was purchased from Merck (Darmstadt, Germany). Methanol was from Honeywell Burdick & Jackson (Muskegon, MI, USA). Water was deionized from a Milli-Q system (Millipore, Bedford, United States).

### 2.2 Samples

The oat (*Avena sativa* L.) powder used in this study was commercial product from Natural Hill (Youngin, Korea). A total of 50 grain products were provided from Rural Development Administration (Jeonju, Korea) in 2022 or 2023. All samples were freeze-dried to achieve less than 3% water content. All samples were stored at −80°C until further analysis.

### 2.3 Parameters on the extraction of lignan

To select the parameters on the extraction of lignan, we select.

#### 2.3.1 Extraction temperature

An amount of 0.1 g of oat powder was weighed in a 2-mL centrifuge tube, and 1 mL of 80% methanol was added and vortexed. Ultrasound sonication was performed at 0°C, 20°C, 40°C, 60°C, and 80°C for 60 min. The extract was then cooled and centrifuged at 22,250 × g at 4°C for 10 min to yield a clear supernatant. The supernatant was filtered through a 0.2 μm nylon filter (Whatman Inc., Maidstone, United Kingdom) into a vial for HPLC-MS/MS analysis.

#### 2.3.2 Solvent concentration

For the extraction, 0.1 g of samples was weighed in a 2-mL microcentrifuge tube, and six different concentrations (water, 20%, 40%, 60%, 80%, and 100% methanol) of methanol were prepared using an ultrasound sonicator at 40°C for 60 min. The sample was then centrifuged at 22,250 × g for 10 min, and the supernatant was filtered through a 0.2 μm syringe filter for HPLC-MS/MS analysis.

#### 2.3.3 Extraction time

The sample (0.1 g) was weighed and transferred to a 2-mL microcentrifuge tube. Then, 1 mL of 80% methanol was added and vortexed for 3 min. Subsequently, the extraction was carried out at 40°C for four different durations (30, 60, 90, and 120 min) using an ultrasonic extractor. Afterward, the extract was filtered through a 0.2 μm nylon syringe filter following centrifugation (22,250 × g, 4°C, 10 min) before HPLC–MS/MS analysis.

### 2.4 Experimental design and statistical model

The RSM was employed to assess the impacts of extraction parameters and optimize conditions for multiple responses. The Box–Behnken design (BBD) was used to determine the extraction parameters for lignans from oats. The experimental design comprised 15 experimental points with three levels (−1, 0, 1) of each factor. The independent variables selected for optimizing lignan extraction were extraction temperature (°C) (X1), methanol concentration (% (v/v)) (X2), and extraction time (min) (X3). The natural and coded values of the factors used in the experimental design are presented in Table 1. The experimental data were fitted to a second-order polynomial model to determine the regression coefficients. The optimum conditions were conducted by 3D response surface plots with the relationship between the independent variables and responses. The second-order polynomial model expressed total lignan contents (μg/100 g) using the following equation:


$$Y=\beta_{0}+\overset{2}{\underset{i=1}{\sum }}\beta_{i}X_{i}+\overset{2}{\underset{i=1}{\sum }}\beta_{ii}X_{i}^{2}+\underset{i=1}{\sum }\underset{j=i+1}{\sum }\beta_{ij}X_{i}X_{j}$$

where Y represents the response variable (total lignan contents μg/100 g), β_0_ is the intercept, β_i_ is the linear regression coefficient, β_ii_ and β_ij_ are the quadratic and interaction terms, respectively. X_i_ and X_j_ are the actual levels of the independent variables. To assess the predictive performance of the model on the response variable, an analysis of variance (ANOVA) was conducted with a confidence level of 95%. The regression coefficient (R^2^) and the *p-*value of the lack of fit were calculated using Minitab ver. 14 software (Minitab Inc., State College, PA, United States).

**Table 1:** Coded and actual levels of three variables in oat.

Independent variables	Coded levels		
	−1	0	1
Temperature (°C) (X_1_)	20	40	60
Methanol concentration (%) (X_2_)	60	80	100
Time (min) (X_3_)	30	60	90

### 2.5 HPLC-MS/MS quantitative analysis of total lignans

The HPLC-MS/MS conditions for total lignans were followed as described in a previous study (43). HPLC-MS/MS analysis was performed using an Agilent 1,260 Infinity II HPLC (Agilent Technologies, California, United States) coupled with an AB Sciex Triple Quad 4,500 LC-MS/MS system (AB Sciex, Framingham, MA, United States) equipped with an electrospray ionization source operating in negative mode. Samples were separated on an Agilent Poroshell C18 column (2.1 × 50 mm, 1.9 μm; Agilent Technologies, United States). The mobile phase A consisted of water, and mobile phase B consisted of acetonitrile, with an injection volume of 2 μL. The separation gradient elution program was as follows: 0–2 min, 15% B; 2–4 min, 50% B; 4–4.1 min, 50% B; 4.1–15 min, 15% B. The flow rate was maintained at 0.4 mL/min, and the column temperature was set at 30°C. The Turbo-V source parameters were configured as follows: curtain gas, 30 psi; collision gas, 9 psi; ion source gas 1, 80 psi; ion source gas 2, 40 psi; ionspray voltage, −4,500 V; ion source temperature, 450°C. The multiple reaction monitoring (MRM) conditions are listed in Table 2. Data acquisition and processing were performed using the AB SCIEX Analyst 1.7.1 Software and MultiQuant (ver 3.0.3).

**Table 2:** MRM transitions and optimized parameters for lignans.

Analytes	RT (min)	Q1 (m/z)	Q3 (m/z)	DP (V)	EP (V)	CE (V)	CEP (V)
Lar	3.39	359.100	329.0	−90	−10	−17	−9
Mat	4.11	356.892	83.0	−70	−10	−50	−7
Pin	3.86	357.000	150.9	−95	−10	−24	−9
Seco	3.25	360.892	164.9	−95	−10	−34	−9
Syr	3.78	416.956	181.0	−80	−10	−26	−9

RT, Retention time; Q1, Parent ions; Q3, Product ions; DP, Declustering potential; EP, Entrance potential; CE, Collision energy; CEP, Cell exit potential; Lar, Lariciresinol; Mat, Matairesinol; Pin, Pinoresinol; Seco, Secoisolariciresinol; Syr, Syringaresinol.

### 2.6 Statistical analysis

Statistical analysis of data was carried out using SAS 9.4 software (Statistical Analysis System, SAS Institute Inc., Cary, NC, United States). The data were expressed as mean ± standard deviation of three experiments. The data were analyzed with one-way analysis of variance followed by Duncan’s multiple range test (*p* < 0.05).

## 3 Results and discussion

### 3.1 Effects of the parameters on the extraction of lignan

The effects of various parameters (extraction temperature, methanol concentration, and extraction time) on the contents of lignans in oats are presented in Table 3. Among the extraction temperatures, the total lignan content of oats ranged from 37.714 μg/100 g to 56.326 μg/100 g, with Syr showing the highest content ranging from 24.454 to 39.592 μg/100 g, while Mat and Seco were not detected. Additionally, the total lignan contents of oats were lowest at 0°C, increased from 20°C to 60°C, and then decreased at 80°C. The total lignan content of oats, using different methanol concentrations, ranged from 18.166 to 56.326 μg/100 g. Among these conditions, the total lignan content was elevated with the use of 80% methanol as the extraction solvent. However, Mat and Seco were not detected. The content of Syr was 18.166 μg/100 g in 0% methanol, while Lar, Mat, Pin, and Seco were not detected. Moreover, Syr was the most abundant in 80% methanol (39.592 μg/100 g), followed by Pin and Lar at 11.252 and 5.482 μg/100 g, respectively. Previous studies reported highly variable ultrasonic extraction times for lignans, ranging from 5 min to 4 h depending on the sample type (25, 44–46). Therefore, this study used extraction times from 30 to 120 min to investigate the effect of extraction time on the lignan content of oats. In this study, most of the lignans showed higher content at 60 min, with a decrease observed at 90 min. The highest total lignan content in oats was observed after 60 min of extraction, with values of 56.326 μg/100 g. However, a significant decrease was observed at 90 and 120 min. Among oats, Syr was the most abundant with 39.592 μg/100 g, while Mat and Seco were not detected. The lignan content depends on the variety of the plant and/or the geographical location of oats. Durazzo et al. (47) studied that Seco and Mat were not detected in oats, which aligns with our results. However, the lignan contents of various oat varieties, with Seco and Mat ranging from 6 to 19 μg/100 g and 0 to 104 μg/100 g, respectively (25). The thermal stability of lignans is known to depend on the structure of the compound and its interactions with other compounds in the plant matrix (44, 48, 49). Gerstenmeyer et al. (49) evaluated that the Lar content in sesame seeds and wheat was stable at 100°C but decreased rapidly above 200°C. These findings indicate that the effect of heat treatment on lignan concentration depends on the plant species, cultivation, extraction, and processing methods (50, 51). Therefore, the results of this study suggest that the differences in oat lignan content depending on temperature are due to differences in oat variety, growing conditions, and extraction methods. Most lignans in grains and seeds are commonly extracted using polar organic solvents (methanol, ethanol, acetone), while less polar lignans can be extracted using non-polar organic solvents such as n-hexane, dichloromethane, and chloroform (5, 18, 52). Additionally, adding 5%–10% water in organic solvents promotes solvent penetration into the plant matrix and facilitates the extraction of polar lignans (5). A previous study examined Seco in flaxseed hulls at various ethanol and methanol concentrations (53). The results showed higher contents of Seco in methanol compared to ethanol, with the highest extraction observed at 70 and 80% methanol concentrations, while extraction yields decreased with ethanol concentrations above 80%. The lignans examined in this study were generally not detected or showed low quantity when extracted with water; however, lignan content increased at 80% methanol compared to 100% methanol. These findings are consistent with previous research, indicating that the five lignans analyzed in this study are more easily extracted in organic solvents than water. Additionally, adding 30% water to methanol improves the polarity of the solvent, facilitating the extraction of polar lignans. Ultrasound-assisted extraction is an approach to create cavitation bubbles within plant cell walls to facilitate solvent penetration and increase the release of various organic compounds (54, 55). The efficiency of the method can be affected by the extraction time, solvent-to-sample ratio, and extraction temperature, which should be taken into consideration (56–58). Previous studies have shown that the lignan secoisolariciresinol diglucoside and phenolic compounds, such as ferulic acid glucoside and caffeic acid glucoside in flaxseed increased with extraction time, specifically more than a 2-fold increase at 60 min compared to 15 min extraction (59). Guo et al. (28) conducted a study to optimize ultrasonic extraction conditions for three lignans ((-)-fargesin, Ses, L-asarinin) from Zanthoxylum armatum roots using RSM. The highest lignan content was observed at 55°C with an extraction time of 40 min, but prolonged sonication time led to its degradation. This study showed that lignan content was highest after 60 min of ultrasonic extraction, with the content decreasing after 90 min. This indicates that the lignans present in the sample matrix were extracted within 60 min, but longer extraction time resulted in ultrasonic degradation of released lignans.

**Table 3:** The effect of different parameters of lignan contents in oats.

Parameters		Lignan contents (μg/100 g)					
		Lar	Mat	Pin	Seco	Syr	Total lignan
Extraction temperature	0°C	3.471 ± 0.175^c^	ND	9.394 ± 0.293^b^	ND	24.848 ± 0.326^c^	37.714 ± 0.208^d^
	20°C	4.995 ± 0.380^ab^	ND	11.376 ± 0.238^a^	ND	25.156 ± 0.873^c^	41.527 ± 0.255^c^
	40°C	5.482 ± 0.046^a^	ND	11.252 ± 0.249^a^	ND	39.592 ± 0.863^a^	56.326 ± 1.157^a^
	60°C	4.788 ± 0.126^b^	ND	9.702 ± 0.258^b^	ND	33.772 ± 1.710^b^	48.262 ± 2.093^b^
	80°C	4.730 ± 0.208^b^	ND	9.811 ± 0.189^b^	ND	24.454 ± 0.760^c^	38.995 ± 1.157^cd^
Methanol concentration	0%	ND^1^	ND	ND	ND	18.166 ± 0.209^d^	18.166 ± 0.209^d^
	20%	1.869 ± 0.007^d^	ND	9.670 ± 0.805^b^	ND	23.380 ± 0.481^c^	34.919 ± 1.293^c^
	40%	2.952 ± 0.326^c^	ND	9.264 ± 0.235^bc^	ND	22.394 ± 0.004^c^	34.610 ± 0.088^c^
	60%	4.090 ± 0.253^b^	ND	8.779 ± 0.157^bc^	ND	26.516 ± 0.313^b^	39.385 ± 0.722^b^
	80%	5.482 ± 0.046^a^	ND	11.252 ± 0.249^a^	ND	39.592 ± 0.863^a^	56.326 ± 1.157^a^
	100%	4.037 ± 0.071^b^	ND	8.587 ± 0.114^c^	ND	26.168 ± 1.054^b^	38.791 ± 1.011^b^
Extraction time	30 min	3.963 ± 0.012^c^	ND	9.060 ± 0.286^c^	ND	25.031 ± 1.020^b^	38.054 ± 0.722^c^
	60 min	5.482 ± 0.046^a^	ND	11.252 ± 0.249^a^	ND	39.592 ± 0.863^a^	56.326 ± 1.157^a^
	90 min	4.710 ± 0.135^b^	ND	10.491 ± 0.470^ab^	ND	25.380 ± 0.059^b^	40.581 ± 0.393^b^
	120 min	4.492 ± 0.484^bc^	ND	10.168 ± 0.081^b^	ND	22.154 ± 0.036^c^	36.814 ± 0.600^c^

All values are mean ± SD. ^a–d^Means with different letters in the same column of each sample are significantly different by Duncan’s multiple range test at *p* < 0.05. Lar, Lariciresinol; Mat, Matairesinol; Pin, Pinoresinol; Seco, Secoisolariciresinol; Syr, Syringaresinol.

### 3.2 Optimization of total lignan extraction conditions

**Figure 2:**
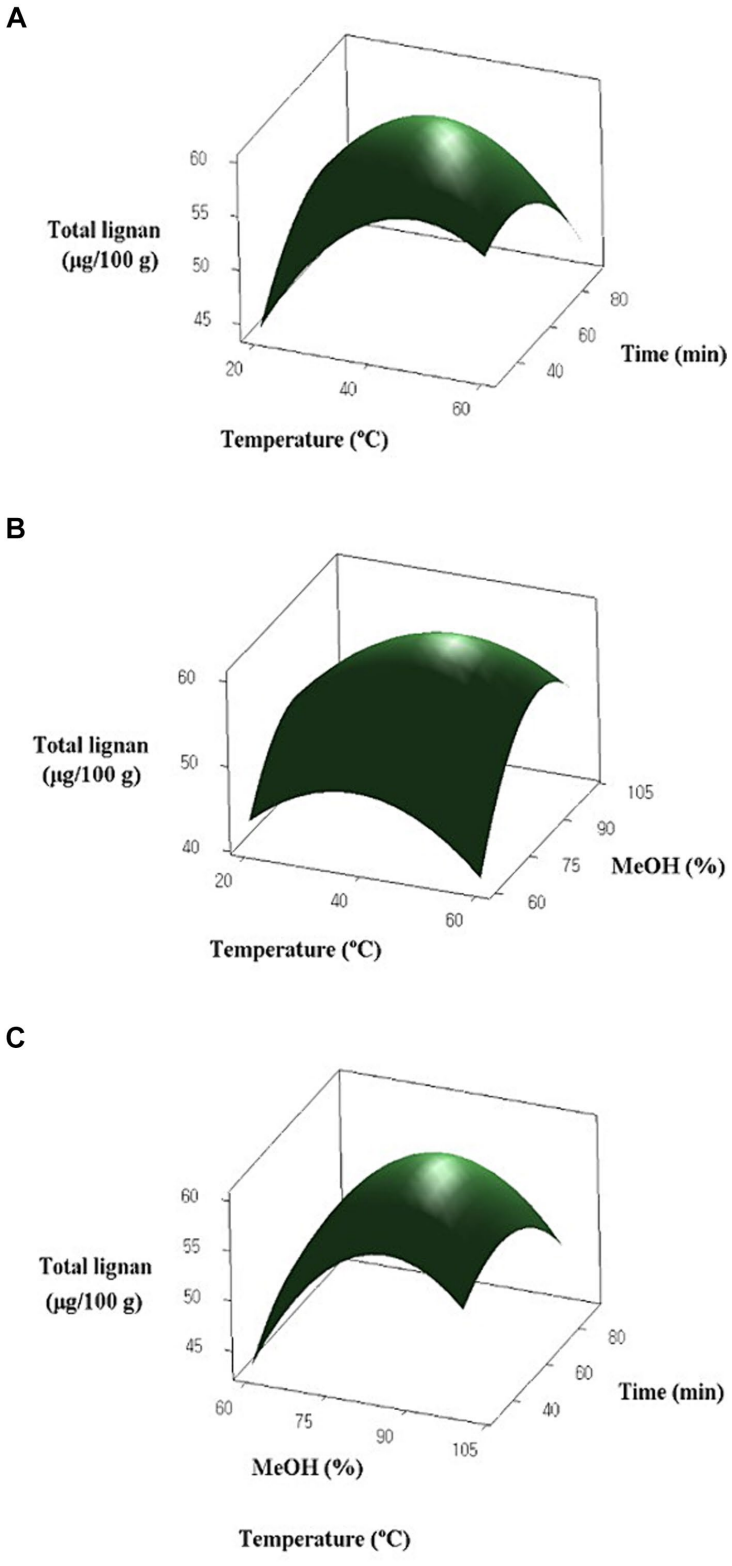
Response surface graphs for the effects of temperature, methanol (MeOH) concentration and extraction time on lignan content of oat: **(A)** Temperature (X_1_) and extraction time (X_3_); **(B)** Temperature (X_1_) and MeOH concentration (X_2_); **(C)** MeOH concentration (X_2_) and extraction time (X_3_).

Fifteen experiments were designed with independent variables of temperature, methanol concentration, and extraction time coded in three levels. The results for the lignan content of oats and the response of the surface are presented in Table 4 and Figure 2, respectively. The lignan content of oats ranged from 38.777 to 60.709 μg/100 g. Among the five lignans, Syr had the highest content, ranging from 23.003 to 41.812 μg/100 g. Pin and Lar were present in the range of 10.494 to 13.025 μg/100 g and 4.041 to 6.346 μg/100 g, respectively, while Mat and Pin were not detected. The experimental results for oats, designed by the BBD, were analyzed for significance and adequacy using ANOVA and are presented in Table 5. The *p*-values of the linear, quadratic, and interaction terms of the model ranged from 0.031 to 0.553, with the term X_2_ acting as the significant term. The linear term, methanol concentration, has a *p-*value of 0.031, indicating a significant effect on the response variable. However, the extraction temperature (X_1_) and extraction time (X_3_) demonstrated *p* > 0.05 and were not influenced by the response variables. Similarly, the quadratic term was found to be a significant variable with a *p-*value of 0.027 in term X_2_, whereas X_1_ and X_3_ did not have a significant effect on the response variable (*p* > 0.05). Furthermore, the interaction term did not show significance in all terms (*p* > 0.05). The results indicated that methanol concentration has a significant effect on total lignan content, whereas extraction time and extraction temperature do not appear to be significant variables. The *p-*value for lack of fit was 0.059, indicating no significant difference, suggesting that the response surface model adequately describes the variation in lignan content. Furthermore, the R^2^ value for the response surface model was 82.39%, indicating a good fit.

**Table 4:** Response surface design and experimental data for the total lignan content in oat.

Run	Variables			Responses in oat (μg/100 g)					
	Temperature X_1_ (°C)	MeOH concentration X_2_ (%)	Time X_3_ (min)	Lar	Mat	Pin	Seco	Syr	Total lignan
1	−1 (20°C)	−1 (60%)	0 (60 min)	5.106	0	10.872	0	26.485	42.464
2	1 (60°C)	−1 (60%)	0 (60 min)	4.733	0	10.610	0	28.044	43.386
3	−1 (20°C)	1 (100%)	0 (60 min)	4.041	0	10.494	0	27.602	42.137
4	1 (60°C)	1 (100%)	0 (60 min)	5.449	0	11.068	0	36.116	52.632
5	−1 (20°C)	0 (80%)	−1 (30 min)	5.416	0	10.859	0	32.977	49.253
6	1 (60°C)	0 (80%)	−1 (30 min)	5.902	0	10.917	0	39.785	56.603
7	−1 (20°C)	0 (80%)	1 (90 min)	6.012	0	11.696	0	30.937	48.645
8	1 (60°C)	0 (80%)	1 (90 min)	4.665	0	10.632	0	25.408	40.705
9	0 (40°C)	−1 (60%)	−1 (30 min)	4.700	0	11.074	0	23.003	38.777
10	0 (40°C)	1 (100%)	−1 (30 min)	5.324	0	13.025	0	31.409	49.759
11	0 (40°C)	−1 (60%)	1 (90 min)	4.817	0	11.922	0	30.984	47.722
12	0 (40°C)	1 (100%)	1 (90 min)	5.364	0	11.563	0	35.385	52.312
13	0 (40°C)	0 (80%)	0 (60 min)	5.854	0	11.526	0	40.444	57.824
14	0 (40°C)	0 (80%)	0 (60 min)	6.346	0	12.220	0	41.812	60.378
15	0 (40°C)	0 (80%)	0 (60 min)	6.608	0	11.703	0	39.203	60.709

Lar, Lariciresinol; Mat, Matairesinol; Pin, Pinoresinol; Seco, Secoisolariciresinol; Syr, Syringaresinol.

**Table 5:** Analysis of variance (ANOVA) of BBD model for total lignan from oat.

Source	DF	Seq SS	Adj SS	Adj MS	*F* value	*p-*value
Model	9	592.302	592.302	65.811	2.60	0.153
X_1_^1)^	1	14.658	66.464	66.464	2.63	0.166
X_2_^2)^	1	74.971	224.108	224.108	8.85	0.031
X_3_^3)^	1	3.137	102.309	102.309	4.04	0.101
$X_{1}^{2}$	1	113.727	151.794	151.794	6.00	0.058
$X_{2}^{2}$	1	221.962	240.504	240.504	9.50	0.027
$X_{3}^{2}$	1	72.270	72.270	72.270	2.85	0.152
X_1_X_2_	1	22.912	22.912	22.912	0.91	0.385
X_1_X_3_	1	58.452	58.452	58.452	2.31	0.189
X_2_X_3_	1	10.213	10.213	10.213	0.40	0.553
Lack of fit	3					0.059
R^2^	82.39%					

^1)^Temperature (°C); ^2)^Methanol concentration (%); ^3)^Extraction time (min). DF, Degrees of freedom; Seq SS, Sequential sum of squares; Adj SS, Adjusted sum of square; Adj MS, Adjusted mean squares.

The regression equation obtained for total lignan is expressed as equation given below:


$$\begin{array}{c} Y_{\mathrm{Total lignan}}=-135.465+1.254X_{1}+3.302X_{2}+1.037X_{3}\\ -\;0.016X_{1}^{2}-0.020X_{2}^{2}-0.005X_{3}^{2}+0.006X_{1}X_{2}\\ -\;0.006X_{1}X_{3}-0.003X_{2}X_{3} \end{array}$$

Table 6 displays the optimized extraction conditions for oat lignans, determined by setting the lignan content to its maximum value. The predicted optimal extraction conditions for oat lignans are a temperature of 44.24°C, a methanol concentration of 84.64%, and an extraction time of 53.53 min. However, in this study, the optimal extraction conditions were modified to include a temperature of 40°C, a methanol concentration of 84%, and an extraction time of 60 min. These parameters were selected for experimental convenience in evaluating the total lignan content of oats. The results showed that the lignan content of the oats was 59.564 μg/100 g, which was similar to the predicted value of 60.213 μg/100 g. Several factors, including solvent-to-solid ratio, solvent concentration, extraction time, and temperature, may significantly impact the extraction efficiency of phenolic compounds from plant sources (60–62). Liyana-Pathirana and Shahidi (63) reported that the concentration of solvent significantly influenced the total antioxidant activity of wheat extracts using RSM. Fombang et al. (64) also reported that solvent concentration had the greatest effect on the total phenol content of *Moringa oleifera* Lam. leaves compared to extraction temperature and extraction time. Additionally, several studies have demonstrated that solvent concentration is an important factor in the extraction of phenolic compounds from various natural products (65–67), consistent with our findings.

**Table 6:** Optimum conditions, predicted and experimental values of responses of oat.

Responses in oat	X_1_	X_2_	X_3_	Total lignan contents (μg/100 g)	
				Predicted	Experimental
Optimum conditions	44.24°C	84.64%	53.63 min	60.213	59.277
Modified conditions	40°C	84%	60 min	-	59.564

X_1_, Temperature (°C); X_2_, Methanol concentration (%); X_3_, Extraction time (min).

### 3.3 Lignan contents of cereal grains

To validate the LC-MS/MS analytical method of lignans, the linearity, limits of detection (LOD), limit of quantitation (LOQ), and intra-day and inter-day precision were evaluated. The linearity was evaluated with six concentration levels (15.625–500 ng/mL) for each analyte, showing acceptable correlation coefficients (R2 > 0.999) (Supplementary Figure 1). The LOD is in the range of 0.041–0.877 μg/100 g, and the LOQ is in the range of 0.118–1.831 μg/100 g (Supplementary Table 1). The intra-day and inter-day precision of the lignans in oats ranged from 0.075%–3.480% and 0.749%–13.735% RSD, respectively (Supplementary Table 2). In this study, a total 53 samples of grains and their products using optimal lignan extraction conditions based on RSM results was analyzed (Table 7). The total lignan contents of the grain samples ranged from 0 to 85.930 μg/100 g. Most of the samples exhibited low lignan content, except for Syr. Barley sprout had the highest total lignan content among the samples with 85.930 μg/100 g. The most abundant lignan in barley sprout was Syr with 41.713 μg/100 g. Lar and Pin were present at 36.491 and 7.726 μg/100 g, respectively, while Mat and Seco were not detected. In wheat, all lignans except Syr were not detected. In addition, the total lignan content of rice increased after cooking process. However, considering that the moisture content of cooked rice is about 65% (68), the content of lignans consumed from cooked rice will be smaller than the value presented in Table 7. For example, the lignan intake from cooked *Saeilmi* brown rice would be 17.648 μg per serving of cooked rice (210 g). Smeds et al. (25) conducted a study on the lignan content of various cereals and found that wheat and barley accounted for approximately 80% of the total lignan content, while oats contained 42% Syr, with higher proportions of Lar and Pin compared to other varieties. The study also showed that Mat was not detected in some wheat and oat varieties, and Lar content varied widely, ranging from 50.3 to 291 μg/100 g depending on the variety. Peñalvo et al. (10) reported that wheat, oat, barley, and rice had the highest levels of Syr, with wheat accounting for over 50% of the total lignan content, three times more than barley and oat. Barley, wheat, and barley had low levels of Mat, ranging from 1 μg/100 g to 3 μg/100 g. The lignan content of barley husks was found to be higher compared to other grains, particularly Lar, which was more than nine times higher compared to other grains. Makowska et al. (18) found that Syr had the highest lignan content among germinated grains, with some varieties showing increased contents of Lar and Syr after germination. Furthermore, Katina et al. (69) reported that germination approximately doubled the lignan levels in wheat. It is known that lignans are more easily released from the structure of germinated grains, leading to an increase in their content. In addition, the breakdown of some starch during germination can increase the content of bioactive compounds such as phenolic compounds. In a study conducted by Gerstenmeyer et al. (49), the content of Lar and Syr increased over time when wheat was steamed at 100°C. The increase in lignan content may be attributed to the facilitation of lignan extraction resulting from changes in the matrix after heating. Therefore, it is considered that the high content of lignan observed in the cooked samples was due to the release of lignans resulting from the gelatinization of starch during cooking.

**Table 7:** The contents of lignan in grains and grain products.

Sample		Lignan contents (μg/100 g dry weight)					
		Lar	Mat	Pin	Seco	Syr	Total lignan
Rice (*Oryza sativa* L.)	*Baegokchal*, white rice, raw	0.398 ± 0.023	ND	ND	ND	3.336 ± 0.006	3.734 ± 0.029
	*Baegokchal*, white rice, cooked	0.931 ± 0.005	ND	ND	ND	3.577 ± 0.036	4.508 ± 0.041
	*Baekjinju*, white rice, raw	0.527 ± 0.035	ND	ND	ND	ND	0.527 ± 0.035
	*Baekjinju*, white rice, cooked	0.682 ± 0.021	ND	ND	ND	ND	0.682 ± 0.021
	*Dodamssal*, white rice, raw	ND	ND	ND	ND	8.527 ± 0.164	8.527 ± 0.164
	*Dodamssal*, white rice, cooked	ND	ND	ND	ND	10.124 ± 0.195	10.124 ± 0.195
	*Haedam*, white rice, raw	ND	ND	ND	ND	7.892 ± 0.253	7.892 ± 0.253
	*Haedam*, white rice, cooked	ND	ND	ND	ND	9.444 ± 0.609	9.444 ± 0.609
	*Heukjinju*, brown rice, cooked	ND	ND	ND	ND	18.642 ± 0.503	18.642 ± 0.503
	*Heukjinju*, brown rice, raw	ND	ND	ND	ND	10.538 ± 0.409	10.538 ± 0.409
	*Odae*, brown rice, raw	ND	ND	ND	ND	9.189 ± 0.026	9.189 ± 0.026
	*Odae*, brown rice, cooked	ND	ND	ND	ND	19.943 ± 1.139	19.943 ± 1.139
	*Odae*, white rice, raw	ND	ND	ND	ND	6.825 ± 0.723	6.825 ± 0.723
	*Odae*, white rice, cooked	ND	ND	ND	ND	8.729 ± 0.197	8.729 ± 0.197
	*Saeilmi*, brown rice, raw	ND	ND	ND	ND	7.805 ± 0.184	7.805 ± 0.184
	*Saeilmi*, brown rice, cooked	ND	ND	ND	ND	24.011 ± 0.683	24.011 ± 0.683
	*Saelimi*, white rice, raw	ND	ND	ND	ND	8.243 ± 0.209	8.243 ± 0.209
	*Saelimi*, white rice, cooked	ND	ND	ND	ND	7.558 ± 0.095	7.558 ± 0.095
	*Samgwang*, brown rice, raw	ND	ND	ND	ND	ND	ND
	*Samgwang*, brown rice, cooked	1.012 ± 0.058	ND	ND	ND	9.080 ± 0.199	10.092 ± 0.141
	*Samgwang*, white rice, raw	1.766 ± 0.095	ND	ND	ND	ND	1.766 ± 0.095
	*Samgwang*, white rice, cooked	1.899 ± 0.028	ND	ND	ND	ND	1.899 ± 0.028
	*Shindongjin*, brown rice, raw	0.980 ± 0.110	ND	ND	ND	6.621 ± 0.112	7.601 ± 0.222
	*Shindongjin*, brown rice, cooked	0.466 ± 0.025	ND	ND	ND	5.858 ± 0.076	6.324 ± 0.051
Rice (*Oryza sativa* L.) products	Instant cooked brown rice	ND	ND	ND	ND	11.342 ± 0.407	11.342 ± 0.407
	Instant cooked white rice	1.859 ± 0.089	ND	ND	ND	1.850 ± 0.016	3.709 ± 0.105
	Instant scorched rice	2.059 ± 0.130	ND	ND	ND	2.895 ± 0.062	4.954 ± 0.191
	Instant rice porridge	1.735 ± 0.037	ND	ND	ND	2.980 ± 0.023	4.715 ± 0.060
	Rice noodle, dried	2.428 ± 0.082	ND	ND	ND	ND	2.428 ± 0.082
	Rice noodle, boiled	3.121 ± 0.147	ND	ND	ND	ND	3.121 ± 0.147
Wheat (*Triticum aestivum* L.) products	Chewy noodle, boiled	ND	ND	ND	ND	8.778 ± 0.091	8.778 ± 0.091
	Chewy noodle, dried	ND	ND	ND	ND	8.452 ± 0.138	8.452 ± 0.138
	Chopped noodle, raw	ND	ND	ND	ND	10.258 ± 0.143	10.258 ± 0.143
	Chopped noodle, boiled	ND	ND	ND	ND	7.293 ± 0.385	7.293 ± 0.385
	Ramyeon noodle, boiled	ND	ND	ND	ND	8.638 ± 0.361	8.638 ± 0.361
	Ramyeon noodle, dried	ND	ND	ND	ND	10.407 ± 0.452	10.407 ± 0.452
	Fine wheat noodle, boiled	ND	ND	ND	ND	7.329 ± 0.072	7.329 ± 0.072
	Fine wheat noodle, dried	ND	ND	ND	ND	8.063 ± 0.313	8.063 ± 0.313
	Spaghetti noodle, boiled	ND	ND	ND	ND	8.266 ± 0.600	8.266 ± 0.600
	Spaghetti noodle, dried	ND	ND	ND	ND	10.006 ± 0.469	10.006 ± 0.469
	Udon noodle, raw	ND	ND	ND	ND	9.437 ± 0.075	9.437 ± 0.075
	Udon noodle, boiled	ND	ND	ND	ND	9.263 ± 0.194	9.263 ± 0.194
	Wheat flour, all-purpose A	ND	ND	ND	ND	11.532 ± 0.267	11.532 ± 0.267	
	Wheat flour, all-purpose B	ND	ND	ND	ND	18.519 ± 1.125	18.519 ± 1.125
	Wheat flour, pan-frying	ND	ND	ND	ND	11.594 ± 0.479	11.594 ± 0.479
Miscellaneous	Barley sprout, dried	36.491 ± 1.928	ND	7.726 ± 0.725	ND	41.713 ± 3.174	85.930 ± 1.970
	Job’s tears (*Coix lacryma-jobi*), raw	ND	ND	ND	ND	8.404 ± 0.390	8.404 ± 0.390
	Job’s tears (*Coix lacryma-jobi*), steamed	ND	ND	ND	ND	10.996 ± 0.363	10.996 ± 0.363
	Oat (*Avena sativa*), *Daeyang*, raw	ND	ND	ND	ND	27.371 ± 0.261	27.371 ± 0.261
	Oat (*Avena sativa*), *Daeyang*, cooked	ND	ND	ND	ND	44.255 ± 1.449	44.255 ± 1.449

All values are mean ± SD. ND, Not detected; Lar, Lariciresinol; Mat, Matairesinol; Pin, Pinoresinol; Seco, Secoisolariciresinol; Syr, Syringaresinol.

In conclusion, optimal extraction conditions of lignans from cereal grains using RSM was investigated. A Box–Behnken design was employed to develop optimal extraction condition of lignan using the three extracting parameters (temperature, methanol concentration, and extraction time). The optimum extract conditions for lignans from grain were obtained at X1 = 44.24°C, X2 = 84.64%, and X3 = 53.63 min. To implement these experimental conditions in the actual experiment, slight modifications were made to the optimal conditions, resulting in X1 = 40°C, X2 = 80%, and X3 = 60 min. The predicted results matched well with the experimental results obtained using the modified optimal extraction conditions. The highest total lignan content of grain was found in barley sprout. These results may be useful for providing reliable data about the lignan contents in cereal grain.

## Data availability statement

The original contributions presented in the study are included in the article/Supplementary material, further inquiries can be directed to the corresponding authors.

## Author contributions

YooK: Formal analysis, Investigation, Writing – original draft. H-WK: Conceptualization, Writing – review & editing. JS: Conceptualization, Supervision, Writing – original draft. YouK: Conceptualization, Funding acquisition, Writing – review & editing.
