# Supplementary material for: Optimal extraction conditions and quantification of lignan phytoestrogens in cereal grains using targeted LC-MS/MS
Source: Front Nutr. 2024 Jun 11;11:1409309. doi: 10.3389/fnut.2024.1409309 (PMC11201688; doi:10.3389/fnut.2024.1409309)
Supplement: Supplementary file 1 [file Data_Sheet_1.docx]

Supplementary Material

# Supplementary Table 1. LOD and LOQ of lignans.

| Analytes | LOD^1)^ (µg/100 g) | LOQ^2)^ (µg/100 g) |
| --- | --- | --- |
| Lar | 0.143 | 0.365 |
| Mat | 0.041 | 0.118 |
| Pin | 0.143 | 0.382 |
| Seco | 0.132 | 0.280 |
| Syr | 0.877 | 1.831 |

# Supplementary Table 2. Intra-day and inter-day precision of LC-MS/MS analysis for lignans.

| Sample | Analytes | Lignans (µg/100 g) | | | |
| --- | --- | --- | --- | --- | --- |
|  |  | Intra-day (n=5)^1)^ | | Inter-day (n=15)^2)^ | |
|  |  | Mean ± SD | RSD  (%) | Mean ± SD | RSD  (%) |
| Oat | Lar | 7.907 ± 0.275 | 3.480 | 6.852 ± 0.941 | 13.735 |
|  | Mat | 0 | - | 0 | - |
|  | Pin | 10.036 ± 0.008 | 0.075 | 11.093 ± 0.949 | 8.554 |
|  | Seco | 0 | - | 0 | - |
|  | Syr | 41.621 ± 1.062 | 2.552 | 41.608 ± 0.474 | 1.139 |
| Total lignan | | 59.564 ± 0.780 | 1.309 | 59.553 ± 0.446 | 0.749 |

^1)^Intra-day refers to the results of 5 independent determinations carried out for the same sample on the same day.

^2)^Inter-day refers to the results of 5 independent determinations carried out on a sample by analyzing 3 replicates of the sample at each day for 5 days.

| (A) Lar | (B) Mat |
| --- | --- |
| 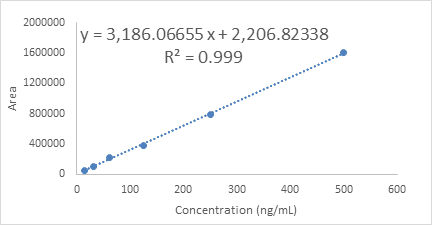 | 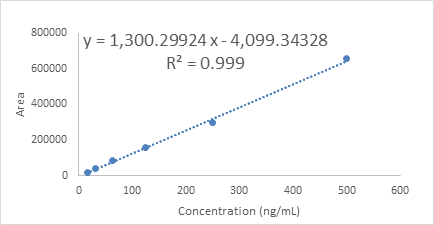 |
| (C) Pin | (D) Seco |
| 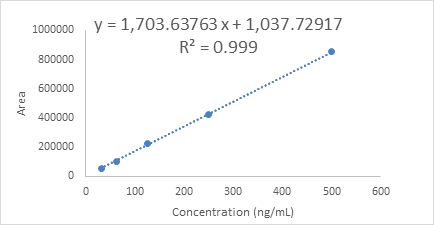 | 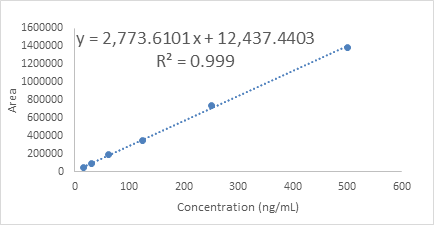 |
| (E) Syr | |
| 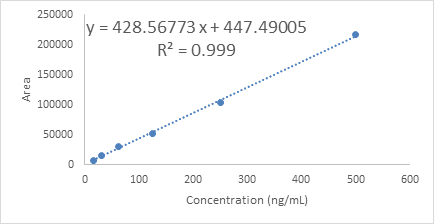 | |

**Supplementary Figure 1.** Calibration curves of lignan standards (A; Lar, B; Mat, C; Pin, D; Seco, E; Syr).
